# A task force for diagnosis and treatment of people with Alzheimer’s disease in Latin America

**DOI:** 10.3389/fneur.2023.1198869

**Published:** 2023-07-11

**Authors:** Francisco Lopera, Nilton Custodio, Mariana Rico-Restrepo, Ricardo F. Allegri, José Domingo Barrientos, Estuardo Garcia Batres, Ismael L. Calandri, Cristian Calero Moscoso, Paulo Caramelli, Juan Carlos Duran Quiroz, Angela Marie Jansen, Alberto José Mimenza Alvarado, Ricardo Nitrini, Jose F. Parodi, Claudia Ramos, Andrea Slachevsky, Sonia María Dozzi Brucki

**Affiliations:** ^1^Grupo de Neurociencias de Antioquia, Universidad de Antioquia, Medellín, Colombia; ^2^Escuela Profesional de Medicina Humana, Universidad Privada San Juan Bautista, Lima, Peru; ^3^Americas Health Foundation, Bogota, Colombia; ^4^Department of Cognitive Neurology, Instituto Neurológico Fleni, Buenos Aires, Argentina; ^5^Internal Medicine, Hospital General San Juan de Dios, Ciudad de Guatemala, Guatemala; ^6^Geriatric Unit, New Hope, Interior Hospital Atención Medica Siloé, Ciudad de Guatemala, Guatemala; ^7^Department of Neurology, HCAM Memory and Behavior Unit, University of Hospital Carlos Andrade Marin HCAM, Quito, Ecuador; ^8^Behavioral and Cognitive Neurology Research Group, Faculty of Medicine, University of Universidade Federal de Minas Gerais, Belo Horizonte, Brazil; ^9^Faculty of Medicine, Department of Functional Sciences, Physiology Division, Universidad Mayor de San Andres, La Paz, Bolivia; ^10^Americas Health Foundation, Washington, DC, United States; ^11^Memory Disorders Clinic, Neurological Geriatrics Program, Department of Geriatrics, National Institute of Medical Sciences and Nutrition Salvador Zubirán, Mexico City, Mexico; ^12^Cognitive and Behavioral Neurology Group, Department of Neurology, Faculty of Medicine, University of Sao Paulo, Sao Paulo, Brazil; ^13^Centro de Investigación del Envejecimiento, Facultad de Medicina, Universidad de San Martín de Porres, Lima, Peru; ^14^Antioquia Neurosciences Group, University of Antioquia, Medellin, Colombia; ^15^Geroscience Center for Brain Health and Metabolism (GERO), University of Chile, Santiago, Chile

**Keywords:** Alzheimer’s disease, Alzheimer’s disease treatment, Latin America, Latin American and Caribbean region, Alzheimer’s disease management, Alzheimer’s disease biomarkers, Alzheimer’s disease recommendations

## Abstract

Alzheimer’s disease (AD) represents a substantial burden to patients, their caregivers, health systems, and society in Latin America and the Caribbean (LAC). This impact is exacerbated by limited access to diagnosis, specialized care, and therapies for AD within and among nations. The region has varied geographic, ethnic, cultural, and economic conditions, which create unique challenges to AD diagnosis and management. To address these issues, the Americas Health Foundation convened a panel of eight neurologists, geriatricians, and psychiatrists from Argentina, Brazil, Colombia, Ecuador, Guatemala, Mexico, and Peru who are experts in AD for a three-day virtual meeting to discuss best practices for AD diagnosis and treatment in LAC and create a manuscript offering recommendations to address identified barriers. In LAC, several barriers hamper diagnosing and treating people with dementia. These barriers include access to healthcare, fragmented healthcare systems, limited research funding, unstandardized diagnosis and treatment, genetic heterogeneity, and varying social determinants of health. Additional training for physicians and other healthcare workers at the primary care level, region-specific or adequately adapted cognitive tests, increased public healthcare insurance coverage of testing and treatment, and dedicated search strategies to detect populations with gene variants associated with AD are among the recommendations to improve the landscape of AD.

## 1. Introduction

Dementia represents a substantial public health challenge in Latin America and the Caribbean (LAC). The demographic structure of LAC has rapidly evolved in recent decades to resemble that of high-income countries (1), with a prevalence of dementia in LAC ranging from 7.1 to 11.5% in individuals over 65 years of age, exceeding that of Europe and the United States. The number of people with dementia in LAC is expected to rise from 7.8 million in 2013 to more than 27 million by 2050, owing to demographic and health changes (2). The widespread idea that dementia and Alzheimer’s disease (AD) are problems in high-income countries must be changed.

AD is the most common cause of dementia in LAC, accounting for 50–84% of cases (3, 4). An aging population and low socioeconomic and educational levels (especially illiteracy) contribute to an increasing prevalence (1, 5). Despite global advancements in fighting dementia, substantial barriers to managing AD persist in LAC. Despite the considerable burden of disease that dementia represents, only Chile, Costa Rica, Cuba, Mexico, and Puerto Rico have national dementia plans; they are insufficiently funded. Argentina discontinued its dementia program (6). The absence of these programs is often due to economic and political instability. Although an exact figure is unavailable, many people living with AD in the region lack access to diagnosis and basic support for their disease. Furthermore, a lack of clinical practice guidelines or recommendations on AD diagnosis and treatment hinders early detection and results in unstandardized management. This review aims to provide multidisciplinary expert recommendations for managing AD in LAC.

## 2. Methods

Americas Health Foundation (AHF) assembled a multidisciplinary panel of eight neurologists, geriatricians, and psychiatrists who are experts in AD from Argentina, Brazil, Colombia, Ecuador, Guatemala, Mexico, and Peru. On September 27, 29, and 30, 2022, they had virtual meetings to develop recommendations for overcoming AD diagnosis and treatment obstacles in LAC. AHF used PubMed, MEDLINE, and EMBASE to identify AD scientists and clinicians from LAC. Augmenting this search, AHF contacted LAC’s medical community thought leaders to confirm that the list accurately represented the required specialties. All the experts who attended the meeting are named authors of this manuscript.

### 2.1. Search strategy

AHF researched AD in PubMed, MEDLINE, and EMBASE. “Treatment,” “management,” “diagnosis,” “quality of life,” and “patient journey” in combination with “Latin America,” and “Alzheimer’s disease” were searched with dates ranging from 01/01/2016 to 06/10/2022. The articles identified were in English, Portuguese, and Spanish. Articles from LAC were prioritized.

Based on the literature search, AHF developed specific questions to address barriers limiting access to AD diagnosis and treatment in LAC and assigned one to each panel member. A written response to each question was drafted by individual panel members based on the literature review and personal expertise. The entire panel reviewed and edited each narrative during the three-day conference through numerous rounds of discussion until a total agreement was reached. An AHF staff member moderated the discussion. When the panel disagreed, additional discussions were held until everyone agreed on the paper’s content. After the conference, seven neurologists, geriatricians, and psychiatrists who are experts in AD from Argentina, Bolivia, Brazil, Chile, Colombia, and Peru reviewed and edited the document. Their contributions were discussed and incorporated. All authors reviewed and approved the final manuscript. The recommendations are based on the evidence gathered and expert opinion and were approved by all authors.

## 3. Results

### 3.1. Landscape of AD in LAC

#### 3.1.1. Burden of disease

Eight population studies conducted in Brazil, Chile, Cuba, Peru, and Venezuela showed a dementia prevalence of 7.1% that doubles every 5 years from 65 years onward (3). The dementia incidence rate was 13.8 per 1,000 people/year for individuals over 65, while that of AD was 7.7 (7). In a systematic review of 17 LAC countries, all-cause dementia prevalence was 10.66%. A higher prevalence was observed among women (8.97%) and rural residents (8.68%). Those with no formal education had more than double the frequency (21.37%) compared to at least 1 year of education (9.88%) (8).

Mortality from dementia increased by 148% between 1990 and 2016. In 2016, dementia was the fifth most common cause of death worldwide, responsible for 2.4 million deaths. Dementia accounted for 4.4% of all deaths and 8.6% of deaths among those over 70, making dementia the second leading cause of mortality in this age group (9). Of 17.4 million people in Chile, there were 3,852 dementia deaths in 2012 (including 1,585 Alzheimer’s deaths) (10). Dementia caused 28.8 million disability-adjusted life years (DALYs) worldwide. It represented 1.2% of DALYs across all ages and 6.3% in those over 70 (11). A study in Chile based on the Chilean national health survey found that dementia had a high impact at the population level in terms of Quality-Adjusted Life Years (QALY) lost and at the individual level in terms of loss of Health-State Utilities (HSU), outnumbering other diseases (12).

### 3.2. Challenges in AD diagnosis and management in LAC

In LAC, the fight against dementia faces pressing challenges, including healthcare access barriers, fragmented healthcare systems, limited research funding, unstandardized diagnosis and treatment, genetic heterogeneity, and varying social determinants of health (5, 11). Additionally, dementia is not a priority for most LAC countries, despite its considerable disease burden (2). Only Chile, Costa Rica, Cuba, Mexico, and Puerto Rico have national dementia plans, but insufficient funding often limits them. Other important barriers are insufficient training in dementia for health professionals and ageism or myths about aging. Four main challenge areas were highlighted at the World Congress of Neurology in Santiago, Chile, in 2015: timely diagnosis, therapeutic approaches and participation in clinical trials, treatment and post-diagnostic support, and research collaboration (6).

There is growing evidence that healthcare-related disparities have a greater impact on populations with low educational and socioeconomic levels, commonly found in LAC. There are often misdiagnoses (13) and delays in referrals to dementia specialists (2). Evidence suggests that racial and ethnic minority groups within LAC are less likely to receive treatment and more likely to discontinue anti-dementia medications (2). Due to fragmentation in healthcare systems, a lack of continuity of care, and access to specialists, patients often remain on antipsychotic medication, even once behavioral symptoms have resolved (14). However, more studies on treatment trends in LAC are necessary. The direct and indirect costs related to AD care are generally borne by the patient’s families, with variations between and within countries and depending on health insurance and socioeconomic level (15, 16).

### 3.3. Region-specific risk factors

Certain risk factors for dementia can potentially be modifiable throughout life. These include low education (8%), hypertension (2%), hearing loss (8%), obesity (1%), smoking (5%), alcoholism (1%), cranial trauma (3%), depression (4%), physical inactivity (3%), social isolation (2%), diabetes (1%) and air pollution (2%). Compared to Europe and North America, the population attributable fraction for modifiable risk factors for dementia is higher due to a greater prevalence of cardiovascular risk factors (17, 18). Of note, these are not risk factors specific for AD but for neurodegeneration and dementia syndrome. In LAC, controlling these factors could prevent up to 40–56% of dementia cases (17, 19, 20).

### 3.4. Research and clinical trials

Research on AD in LAC has grown recently but is still limited, mainly due to restricted funding. In 2013, there were 715 AD clinical trials globally, and only 34 were in South America (21). By 2020, only 6% of active trials included LAC countries, mainly Argentina, Brazil, Chile, Colombia, and Mexico (6). LatAm-FINGERS, the dementia prevention lifestyle-change trial for dementia prevention, has been operating since 2021. This is the only dementia trial designed in Latin America and brings together 12 countries in the region (Argentina, Brazil, Bolivia, Chile, Colombia, Costa Rica, Ecuador, Mexico, Peru, Puerto Rico, Dominican Republic, and Uruguay) (22). Scarce regional data impacts understanding pharmacokinetics, pharmacodynamics, and genetic influences and hinders public policy development. Favorable regulatory environments for clinical trials, clinical trial unit infrastructure, and increased funding mechanisms must be developed to increase LAC participation in AD research.

### 3.5. Diagnosis of AD in LA

LAC countries have different cultural, linguistic, and ideological nuances determining AD detection, management, and prognosis. The main obstacles to early AD detection in LAC are misconceptions surrounding memory impairment, training at the primary care level, and specialist availability (23).

AD diagnosis in LAC is primarily based on clinical information and cognitive tests. Biomarkers and imaging are limited to a few centers, and genetic screening is rare (9). When applied effectively, clinical criteria (24, 25) when used effectively, may offer up to 90% diagnostic accuracy. Some countries have recommendations and guidelines for dementia diagnosis and management (Argentina (26), Brazil (27), Chile (28, 29), Colombia (30), Mexico (31), Peru) (23, 32, 33).

According to DSM-5, dementia includes memory loss, cognitive decline, and impairment of functionality. Consequently, these must be evidenced in clinical practice using brief cognitive tests (BCTs) and questionnaires about activities of daily living (ADL). Mini-Mental State Examination (MMSE), Montreal Cognitive Assessment (MoCA), Addenbrooke’s cognitive examination (ACE III), and Consortium to Establish a Registry for Alzheimer’s Disease (CERAD) are the most widely used instruments across LAC (8). Evidence shows sociodemographic variables, including age, sex, education, literacy, and language impact BCT performance (32). The high prevalence of lower educational achievement and socioeconomic disparities in LAC (34) and the fact that the performance of illiterate individuals on neuropsychological tests often resembles that of literate individuals with dementia may contribute to misdiagnosis (8, 35, 36). Thus, as a minimum, countries should have cognitive tests validated for their local context. The Rowland Universal Dementia Assessment Scale (RUDAS) (35) and Brief Cognitive Screening Battery (BCSB) (36, 37) could better adapt to the conditions of LAC populations. The Pfeffer Functional Activities Questionnaire (PFAQ) and the Technology-Activities of Daily Living Questionnaire (T-ADLQ) are recommended to evaluate ADL (38).

**Figure 1:**
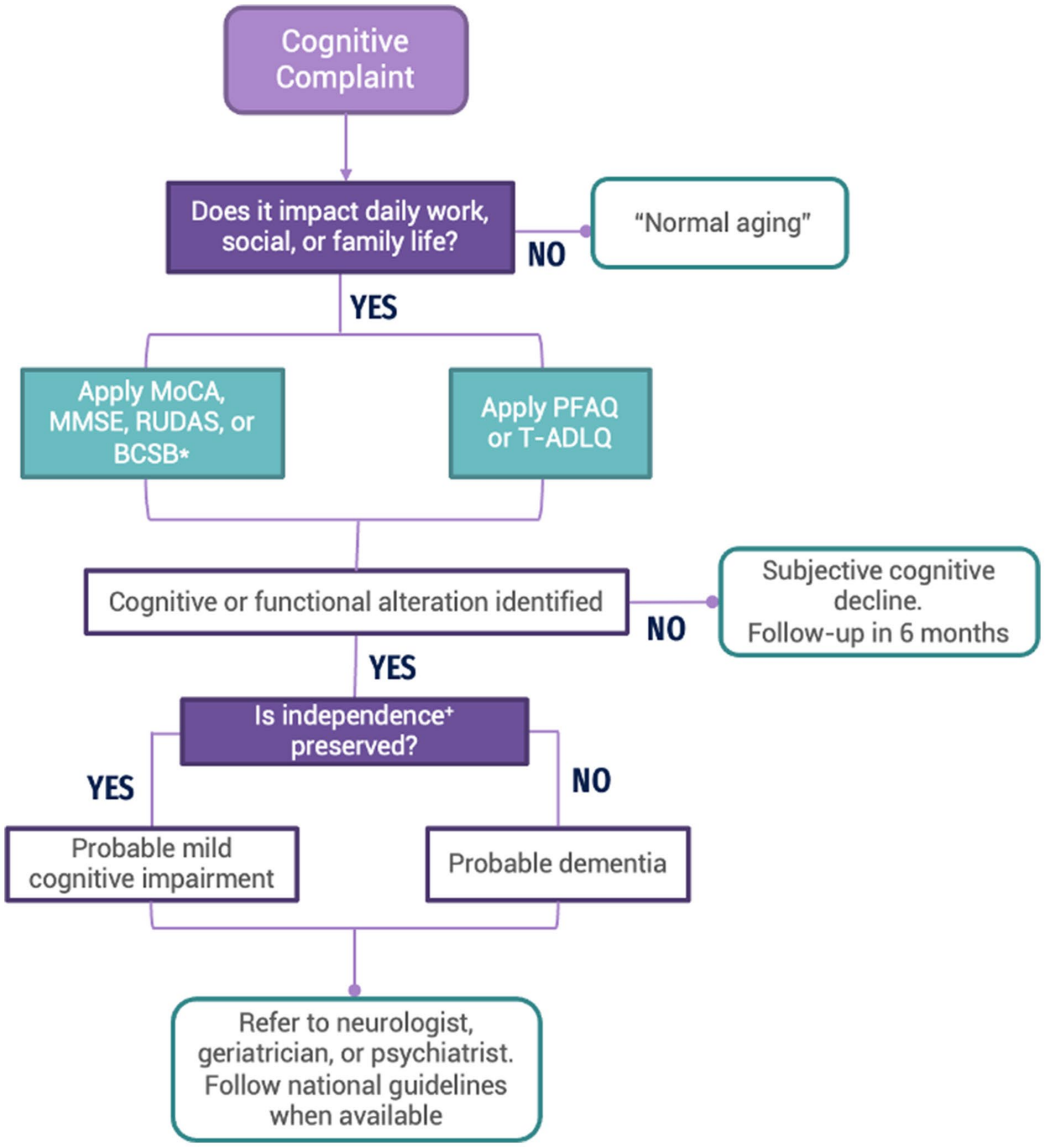
Flow chart depicting the recommended process to assess patients with cognitive complaints at the primary care level in LAC. MoCA, Montreal Cognitive Assessment; MMSE, Mini-Mental State Examination; RUDAS, Rowland Universal Dementia Assessment Scale; BCSB, Brief Cognitive Screening Battery; PFAQ, Pfeffer Functional Activities Questionnaire; T-DALQ, Technology-Activities of Daily Living Questionnaire. *Adjusted for patient’s age, literacy, and educational levels. ^+^Independence is understood as the ability or aptitude to make informed and responsible decisions in ADL (managing financial affairs, fulfilling a desired task, driving a vehicle, administering medications, or living independently) (40).

Many communities in LAC only have access to primary care. Opportunities for diagnosis may be missed because primary care providers (PCPs) often fail to screen older adults for AD due to insufficient time and training. Early symptoms of dementia, such as memory impairment, may not be apparent during a routine office visit unless they are directly assessed (39). Thus, it is crucial to increase LAC PCPs’ awareness of their role in AD early detection and training on the appropriate application of cognitive assessment tools. To this end, recommendations are proposed for evaluating memory complaints at the primary care level in LAC (See Figure 1).

#### 3.5.1. Assessment of patients with cognitive complaints at the primary care level in LAC

1. A complete anamnesis and physical exam should be conducted. The presence of an accompanying family member or caregiver is necessary. A structured interview should identify whether the cognitive complaint affects the patient’s daily work, social or family life. If the answer is yes, the evaluation should be continued. Notably, questions about behavioral or emotional symptoms should be asked.
2. Conduct a cognitive evaluation using MoCA, MMSE, RUDAS, or BCSB (41) with the appropriate adjustments based on the patient’s age, education, and literacy level.
3. Conduct a functional evaluation using the PFAQ or T-ADLQ: Technology–Activities of Daily Living Questionnaire’.
4. If an impairment is identified in cognitive or functional assessments, refer to a specialist (neurologist, geriatrician, or psychiatrist) for continued evaluation and care or follow the national guideline for dementia.

#### 3.5.2. Evaluation of a patient with a suspected cognitive deficit at the specialist level in LAC

1. Conduct a complete physical examination and a direct interview to understand the patient’s context and support network, comorbidities, current medication, and the level of impact of symptoms on the patient’s life. The presence of a family member or caregiver is necessary. Consider differential diagnosis (Table 1).
2. Order a formal neuropsychological evaluation (if available), particularly if symptoms are mild [e.g., mild cognitive impairment (MCI)] or in cases where the differential diagnosis is challenging (e.g., non-amnestic/atypical presentations of AD).
3. Order additional testing. These may include imaging studies. The neuroimage of choice in patients with dementia is magnetic resonance imaging (MRI). In cases where it is unavailable or contraindicated, a computerized tomography (CT) can be performed (suggested neuroimaging can be found in Table 2). Laboratory tests: thyroid profile, lipid profile, vitamin B12, folic acid, venereal disease research laboratory test (VDRL), blood glucose, renal function, complete blood count with sedimentation rate, and electrolytes must also be ordered to rule out reversible causes of cognitive impairment or clinical comorbidities that may impair cognitive functioning.
4. Make treatment and follow-up decisions based on evaluation findings.
5. Order biomarkers, when available, for patients with symptom onset below 65 years of age or MCI or atypical AD clinical presentations.

**Table 1:** Differential diagnosis for AD in LAC (42–44).

Differential diagnosis	Differentiating characteristics
Chronic subdural hematoma	History of traumatic brain injury is not always present. Progressive symptoms of headache, changes in mental status, weakness, gait disturbance, lack of sphincter control, and if it is not treated, it can evolve into nausea, vomiting, coma, and death.
Normal pressure hydrocephalus	Typical features are progressive hydrocephalus on imaging and a triad of dementia, magnetic gait (apraxic gait), and incontinence.
Dementia due to nutritional deficiency	Memory impairment, personality changes associated with loss of vibration and proprioception, spastic paraparesis, and neuropathy.
Vascular dementia	Secondary to occlusion of large- or small-vessel disease; to arteriolopathy, causing a progressive or stepwise impairment; or hemorrhagic causes.
Frontotemporal dementia	Clinical spectrum encompasses the bvFTD, the semantic variant of primary progressive aphasia (PPA), and the nonfluent or agrammatic variant of PPA. BvFTD is characterized by early and prominent personality and behavioral changes (apathy, loss of empathy, social isolation, lack of motivation, stereotyped or compulsive behavior, and executive deficits).
Atypical Parkinsonism	Progressive supranuclear palsy and corticobasal syndrome are commonly grouped in the FTD spectrum.
Dementia with Lewy bodies	Characterized by alpha-synuclein accumulation. Dementia develops at least one year after the onset of parkinsonism. The more common symptoms are recurrent visual hallucinations, fluctuations in attention and alertness, declining cognitive abilities such as problem-solving, increased visuospatial problems, and frequent REM sleep disorder.
Chronic infectious diseases	Neurocysticercosis, syphilis, tuberculosis, HIV infection can cause a progressive course, often with CSF alteration. They are potentially treatable dementias. Syphilis commonly causes a frontal dysexecutive syndrome and psychiatric symptoms (psychosis, hallucinations, depression, and mania).
Prion dementia	Characterized by its rapid progression due to abnormal prion protein deposits. Clinically presents with myoclonic movements and ataxy.
Cognitive impairment due to depression or medications	Can be due to depression or medication. It can be reversible by treating depression or removing the medication causing the undesirable effect.

bvFTD, behavioral variant of frontotemporal dementia; PPA, primary progressive aphasia; FTD, frontotemporal dementia; REM, rapid eye movements; CSF, cerebrospinal fluid; REM, rapid eye movement.

**Table 2:** Magnetic resonance imaging protocol for patients with dementia (45).

Magnetic resonance imaging protocol for patients with dementia
1. 3D-T1 Isotropic Sequence with 1.2–1.4 mm voxel (5–7 min).
2. FLAIR Sequence (2D axial 4–5 mm or sagittal 3D isotropic) (3–5 min).
3. T2* Sequence 3–5 mm axial (1 min).
4. Axial diffusion (b 1,000) (1 min).
5. Coronal T2 3 mm.
6. Optional: ASL perfusion.

ASL, arterial spin labeling; FLAIR, fluid attenuated inversion recovery.

#### 3.5.3. Alzheimer’s disease staging and classification

Given that the pathophysiological processes of AD are a continuum that begins years before the first symptoms appear, the US National Institute on Aging and the Alzheimer’s Association proposed two AD diagnostic categories based on their stage of progression: preclinical or asymptomatic (biological definition of AD); and clinical or symptomatic (clinical definition of AD). Of note, preclinical diagnosis is currently recommended only in research contexts. The clinical stage can, in turn, be divided into two subcategories: MCI; and dementia in mild, moderate, and severe stages. AD can be monitored throughout this continuum using biomarkers or direct and indirect indicators of neuropathological changes in the brain, as well as clinical signs and symptoms (46). In LAC, monitoring with biomarkers and indicators of neuropathological changes in the brain is currently applied primarily in research contexts.

**Figure 2:**
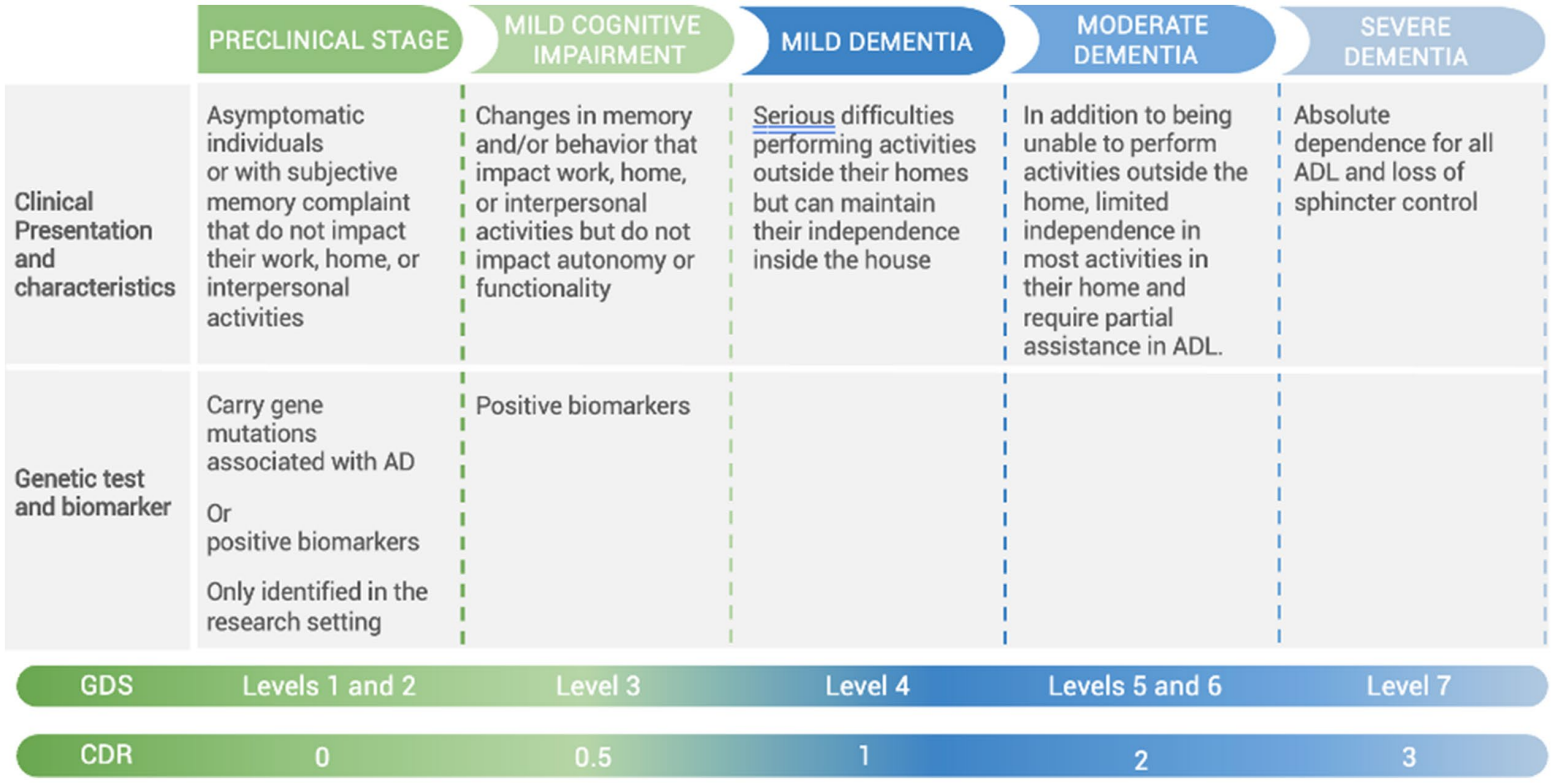
Correlation of AD clinical stages with Global Deterioration Scale (GDS) and Clinical Dementia Rating Scale (CDR). ADL, activities of daily living; AD, Alzheimer’s disease.

The preclinical stage can also be subdivided into stages. This has been done with the genetic form of AD caused by the Paisa mutation (E280A in *PS1*). This mutation affects 25 multigenerational families in Mendelian inheritance, representing the largest group in the world, consisting of more than 6,000 inheritors and 1,200 carriers. The time between birth and 24 years of age is Phase 0, when abnormally high levels of pTau217 and neurofilament light chain (NfL) are detected in plasma and low levels of Aß42 -amyloid in cerebrospinal fluid (CSF). Phase 1 lasts from 24 to 28 years when amyloid-positive cells begin to appear on the positron emission tomography (PET)-amyloid image. Phase 2 is from 28 to 32 years of age when a significant reduction in memory scores on the 10-word CERAD list is detected in asymptomatic people without memory problems. In stage 3, from 32 to 38 years of age, positive signs of tauopathy appear in the PET-tau image, and subjective memory loss occurs without impact. Finally, in Phase 4, from 38 to 44 years of age, when memory complaints with impact and MCI appear (47, 48). The most used staging scales for AD in LAC are the Global Deterioration Scale (GDS) and Clinical Dementia Rating Scale (CDR); GDS offers significant advantages by more clearly defining the MCI stage, in addition to having validation studies in LAC (46). The correlation of AD clinical stages with the GDS and CDR is shown in Figure 2 (1, 49, 50).

### 3.6. Non-pharmacological management of AD

After a patient receives a diagnosis, the pathway to receive treatment and support varies among countries and depends widely on what type of healthcare insurance the patient has. Non-pharmacological management encompasses a wide range of non-invasive, safe approaches and techniques that, when used in a standardized and guided manner, can improve cognitive function, neuropsychiatric symptoms, and independence in patients with AD, as well as their caregivers’ quality of life (QoL). These interventions do not influence the underlying pathophysiological mechanisms; they focus on maintaining the patient’s functionality for as long as possible as the disease progresses (51). A scoping review was conducted of non-pharmacologic interventions for caregivers of people living with dementia in LAC (52), but there are scarce publications about non-pharmacological strategies aimed at the patient residing in any LAC country (53). Three months of multimodal training contributed to mobility and executive function in elderly individuals with mild cognitive impairment but not in those with Alzheimer’s disease; additionally, in Massachusetts, an intervention (Virtual mentalizing imagery therapy) has been adapted for Hispanic participants living in Boston (54).

#### 3.6.1. Exercise and motor rehabilitation

Multiple studies confirm the positive effect of exercise programs in reducing the progression of dependence in patients with AD (55–57). In animal models, exercise therapy improved cognitive performance mechanisms, such as increasing growth factors (58), alleviating oxidative stress (59), decreasing antibody concentrations (60). Early intervention in the 3xTg-AD mice with an amyloid β-antibody fragment ameliorates the first hallmarks of Alzheimer’s disease (60), and inhibits tau phosphorylation from slowing the progression of dementia (60). Treadmill exercise promotes E3 ubiquitin ligase to remove amyloid β and P-tau and improve cognitive ability in APP/PS1 transgenic mice (61), but the applicability in humans may have some variability in cognitive improvement due to the differences in exercise modality, intensity, frequency, and duration of study design; so, future research on the process of exercise therapy for cognitive improvement needs to describe more specific exercise modalities and find more accurate ways to mitigate the process of dementia (62).

#### 3.6.2. Cognitive stimulation

On the other hand, concerning rehabilitation goals and collaborative goal-setting in patients with mild-to-moderate dementia was feasible, especially when patients were supported by a structured approach, which yielded a more holistic view of potential rehabilitation goals and needs from the patients’ perspective. Mobility-related functions were stated as the most critical rehabilitation goals, followed by functions related to psychological well-being, and self-reported functional problems showed a significant relationship with objective clinical assessments indicating a sustained insight into acute functional deficits in patients with mild-to-moderate dementia (63). Cognitive stimulation involves themed activities and is typically implemented twice weekly. Several studies report improved general cognitive functioning in patients with mild-to-moderate dementia (7, 64). Current evidence suggests that it can be delivered face-to-face or online (65, 66). Integrating exercise, proper nutrition, and cognitive and social interventions may significantly mitigate cognitive decline in people with dementia. In fact, the benefits of non-pharmacological interventions provide the basis for future pharmacological interventions (67).

#### 3.6.3. Non-pharmacological management of behavioral and psychological symptoms of dementia

All guidelines agree that the first-line treatment for behavioral and psychological symptoms should be non-pharmacological management. Interventions range from tailored activity programs to sensorial, psychological, and behavioral approaches, including environmental redesign, validation, reminiscence, and light therapy (7, 68). A first-line pharmacological approach should only be used if neuropsychiatric symptoms cause a high risk of harm (6).

Other types of non-pharmacological therapy, such as occupational therapy, including activity simplification, adaptive aids, problem-solving strategies, skill training, and caregiver education, have shown slight improvements in patient and caregiver QoL (69). Complementary therapies such as transcranial magnetic stimulation, alternative medicine, music, art, aroma, and massage therapy require further study to determine their benefit (7). Preliminary studies on communication technology therapies, such as home automation, virtual reality, video games, and telemedicine, show promising results that have yet to be confirmed (7).

### 3.7. Pharmacological therapy

Pharmacological therapy for AD currently consists of symptomatic treatments that seek to lessen the impact of cognitive symptoms, alleviate behavioral and psychological symptoms, and preserve functionality. There is great heterogeneity in the availability of anti-dementia treatments in LAC. In some countries, they are available through private or public healthcare; in others, they entail out-of-pocket costs for patients and their caregivers (1) (Table 3).

**Table 3:** Characteristics and access of cognitive-enhancing medications approved for AD.

Characteristics	Donepezil (70)	Rivastigmine (71, 72)	Galantamine (73)	Memantine (74)
Mechanisms of action	Noncompetitive, rapidly reversible AChE inhibition (3, 4)	Noncompetitive, slowly reversible BuChe and AChE inhibition (3, 4)	Competitive, rapidly reversible AChE inhibition (3, 4)	Low-affinity, voltage-dependent, noncompetitive antagonist of the NMDA receptor
Formulations	Tablets	Capsules Oral solution Transdermal patch	Capsules Oral solution	Tablets Oral solution (4)
Indications	Mild, moderate, severe AD	Mild-to-moderate AD, Parkinson’s disease dementia	Mild-to-moderate AD	Moderate to severe AD- combined with AChE may have additional efficacy
Recommended dose	Oral immediate release & Disintegrating tablet: Initial dose 1.5 mg QD Increase after 1 month to maintenance dose 10 mg QD	Oral: Initial dose 1.5 mg bid with Meals Increase by 3 mg daily Q2 week to maintenance dose of 6 mg bid with meals Transdermal patch: Initial dose: 4.6 mg patch QD Increase no sooner than 4 weeks to 9.5 mg patch, then max dose of 13.3 mg patch QD Rotate patch site	Oral immediate release/ solution: Initial dose 4 mg BID with meals Increase by 8 mg QS q4 weeks to a maintenance dose of 12 mg bid Oral extended release: Initial dose 8 mg QD Increase to 16 mg QD Q4 weeks to maintenance dose of 24 mg QD	Tablets/ Oral solution Titration Week 1: 5 mg QD Week 2: 5 mg BID Week 3: 10 mg qAM, 5 mg QHS Week 4 and after: 10 mg bid or 20 mg QD when available Available in combination w/donepezil
Most common adverse reactions occurring at a frequency of at least 5%	Nausea, diarrhea, insomnia, vomiting, muscle cramp, fatigue, anorexia	Oral: Nausea, vomiting, diarrhea, anorexia, dyspepsia, asthenia Patch: Allergic dermatitis, Nausea, vomiting, diarrhea. Usage shows reduction in nausea, vomiting, asthenia (*p* < 0.001 vs. placebo), headaches, weight loss, and dizziness (p < 0.001 vs. oral) (2)	Nausea, vomiting, diarrhea, dizziness, headache, decreased appetite, weight loss	Dizziness, headache, confusion, constipation
Availability in the country	Available in Argentina, Brazil, Chile, Colombia, Ecuador, Guatemala, México, Perú, Bolivia	Available in Argentina, Brazil, Chile, Colombia, Ecuador, Guatemala, México, Perú, Bolivia	Available in Argentina, Brazil, Chile, Colombia, Ecuador, México, Perú	Available in Argentina, Brazil, Chile, Colombia, Ecuador, Guatemala, México, Perú, Bolivia
Covered by public healthcare system	Argentina: YES * Bolivia: NO Brazil: YES Chile: YES^+^ Colombia: YES Ecuador: NO Guatemala: NO México: YES*** Perú: NO	Argentina: YES * Bolivia: NO Brazil: YES** Chile: YES^+^ Colombia: YES Ecuador: NO Guatemala: YES, oral solution; NO, patch Mexico: YES*** Peru: NO	Argentina: YES * Bolivia: NO Brazil: YES^+^ Chile: YES Colombia: YES Ecuador: NO Guatemala: NO México: YES*** Perú: NO	Argentina: YES * Bolivia: NO Brazil: YES^+^ Chile: YES Colombia: YES Ecuador: NO Guatemala: NO México YES*** Perú: NO

AChEI, acetylcholinesterase inhibitor; AChE, acetylcholinesterase; BuChe, butyrylcholinesterase; AD, Alzheimer’s disease; mg/d, qd, once-daily; bid, twice daily; qAM, once-daily in the morning; q 4 weeks, every 4 weeks; *In Argentina, the national disability law (law 22,431, 24,901) determines the possibility of accessing a “Certificado Unico de Discapacidad” (CUD) (75), or unique disability certificate that mandates 100% coverage of all medical treatment, including medication, devices, treatments, and transport, among others. AD is included within this law in all healthcare systems, so all treatments for AD are covered. However, families must undergo a process to obtain the certificate and present it to the “Programa de Atencion Médica Integral (PAMI)” (76) or Medical care program for older adults. Often, families are unaware of this access route or do not undergo the process, especially in low-resource settings.

**13.3 mg patch not covered by the Sistema Unico de Saude (SUS). ***All four medications are covered by the (INSABI), but they are not always available for dispensing. ^+^Included in AUGE (universal health plan which provides explicit healthcare guarantees) and must be prescribed by a specialist.

Of the four FDA-approved drugs for AD treatment, three are acetylcholinesterase inhibitors (AChEIs) (donepezil, galantamine, rivastigmine) (2) and one, memantine (3), is an N-methyl-D-aspartate (NMDA) receptor antagonist. In some LAC countries, molecules approved for commercial use are often prescribed as adjuvants in AD treatment. These include *Ginkgo biloba*, cerebrolysin, nimodipine, and citicoline. All of them lack scientific evidence for their use in AD.

Rivastigmine patches have additional benefits, including simple administration, once-daily application, caregiver empowerment, prevention of accidental overdose, small and discrete size, comfort, and immediate removal in an emergency (77). From the pharmacological point of view, patches allow sustained plasma levels, reduce adverse events, and allow easier access to the optimal dose. Moreover, they avoid the digestive tract, making them independent of food intake and preventing first-pass loss (77, 78).

### 3.8. Recommendations for the management of AD

To improve treatment adherence, patients and caregivers should be counseled on realistic treatment outcomes and expectations of stabilizing cognitive symptoms and improving QoL rather than achieving noticeable improvements.

#### 3.8.1. The prevention and management of AD within an integrated and person-centered health services framework

What “matters to the person” is one of the factors that should guide the care and design of health services in the 21st century (79–81). That means person center care versus disease center care. Early detection of cognitive impairment and AD can take several paths in LAC. The Pan American Health Organization has defined healthy aging as the process of greater functional ability that allows living with well-being. The functional ability (“to be and do what one considers valuable”) comprises the intrinsic capacity (sum of physical and mental capabilities), the environment, and the interaction between the two. The intrinsic capacity has several components or domains (cognitive, psychological, sensory, mobility, and vitality). Persons are integral beings, and the alteration in one of the domains of intrinsic capacity can be a warning sign of a latent or potential cognitive alteration (82). Bidirectional associations of the cognitive domain with mobility (83) (cognitive motor syndrome (84), cognitive frailty) (85), sensory (visual (86) and hearing (87)), psychological (88), and vitality (89)(metabolism and nutrition) have been demonstrated. Likewise, the manifestations in different domains worsen the prognosis of active life and well-being (90). Detecting changes in any of these domains can help detect early and manage cognitive alterations that could materialize in AD or its risk factors (91). At the same time, managing alterations in domains other than cognitive can optimize cognitive performance and facilitate care (92, 93). One of the justifications for the early diagnosis of AD is that the person can decide on treatment options at all stages of the disease (94).

#### 3.8.2. Mild cognitive impairment corresponding to GDS level 3

**Figure 3:**
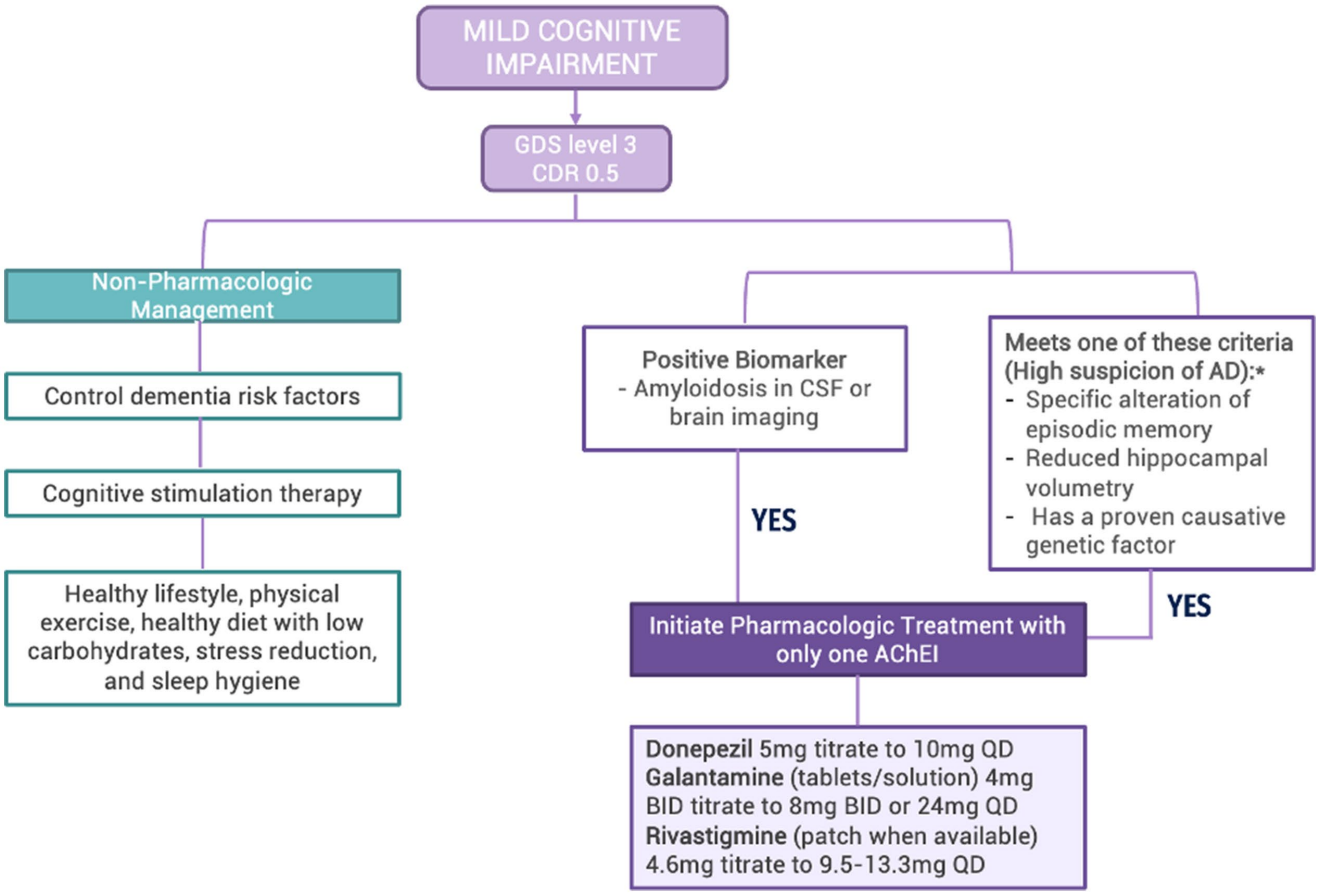
Algorithm for the management of mild cognitive impairment in LAC Legend: GDS: Global Deterioration Scale; CDR: Clinical Dementia Rating Scale; CSF: cerebrospinal fluid; AD: Alzheimer’s Disease; AChEI: acetylcholinesterase inhibitors *Although AChEI is not currently approved for the indication of MCI, this expert panel recommends its use in the specified situation.

Although AChEI are not approved for MCI in LAC, they are commonly prescribed for this indication, according to this panel’s experience. Some evidence exists of slight cognitive benefits and delays in conversion to dementia using donepezil in people with MCI (95, 96). This panel recommends initiating therapy with AChEI if there is amyloidosis in CSF or brain imaging or if there is a high suspicion of MCI due to AD because the patient meets one or more of the following criteria: (1) specific alteration of episodic memory; (2) reduced hippocampal volume; (3) has a proven causative genetic factor. Of note, memantine is often inadequately initiated at diagnosis, even if the patient has MCI or mild dementia. This is an erroneous practice, which should be avoided as memantine is not indicated in these cases (See Figure 3).

#### 3.8.3. Mild–moderate dementia due to AD corresponding to GDS levels 4–5

**Figure 4:**
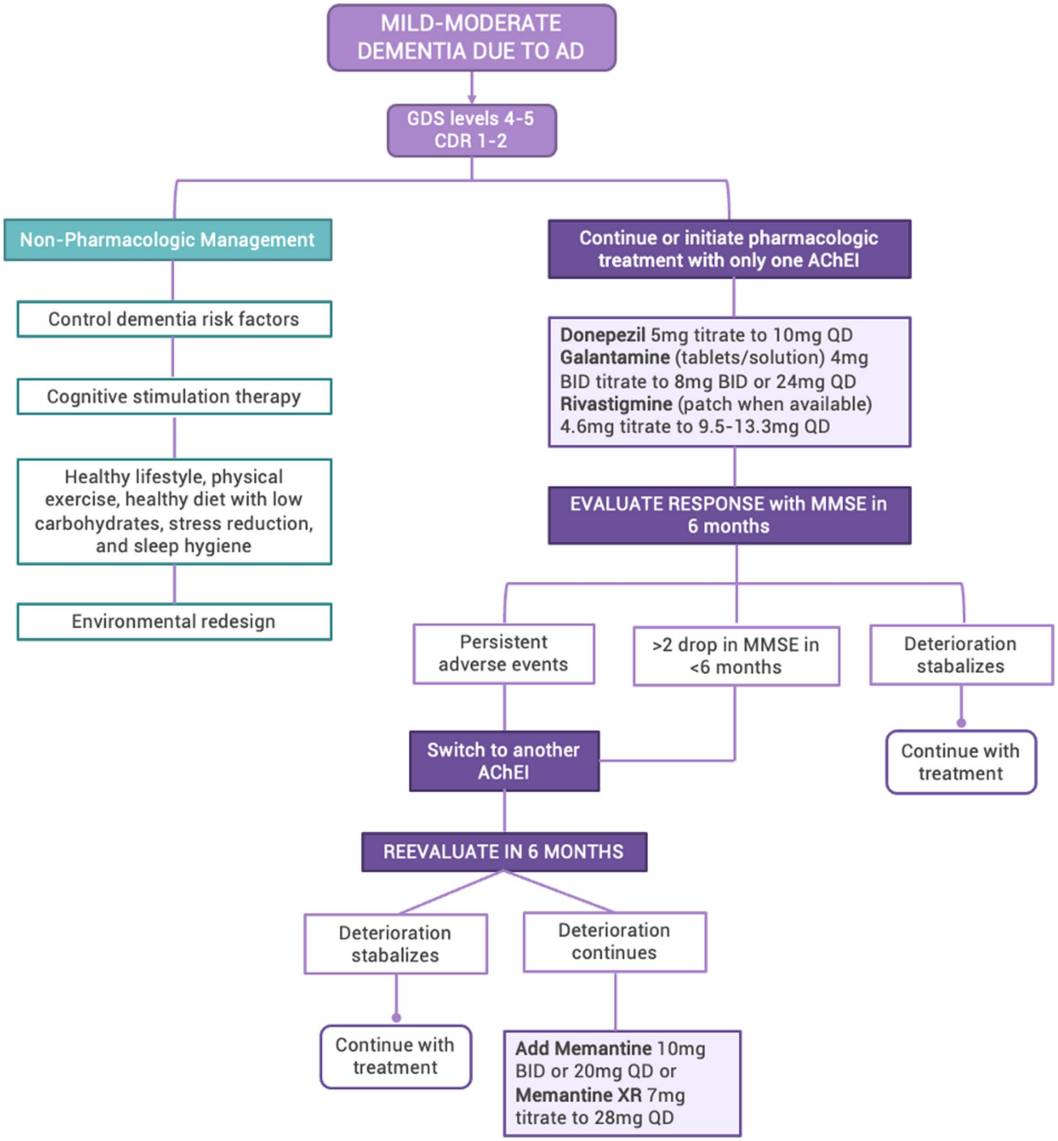
Algorithm for the management of mild–moderate dementia due to AD in LAC GDS, Global Deterioration Scale; CDR, Clinical Dementia Rating Scale; AD, Alzheimer’s Disease; AChEI, acetylcholinesterase inhibitors; MMSE, Mini-Mental State Evaluation.

For this group, treatment and management based on the following principles are required (See Figure 4):

1. Non-pharmacological interventions (97–99): A healthy lifestyle with physical exercise, a healthy diet with low carbohydrates, stress reduction, and sleep hygiene; control of dementia risk factors; cognitive stimulation therapy; and environmental redesign.
2. Pharmacologic therapy (100): Using one AChEI that increases the level of acetylcholine; if deterioration continues, measured by a drop >2 points on the MMSE in <6 months, a different AChEI should be initiated. If deterioration continues, memantine should be added to the treatment regimen.
3. Participation in pharmacological and non-pharmacological tertiary prevention studies, pending the commercial availability of these treatments, or palliative studies, depending on disease progression.

#### 3.8.4. Moderately-severe and severe dementia due to AD corresponding to GDS levels 6–7

Severe behavioral and psychological symptoms are hallmarks of this stage, and many patients require long-term institutional care (101, 102, 103). In LAC, most caregivers of patients with AD are women, frequently wives or daughters, that co-reside with the patient and do not receive financial compensation (104, 105). Severe AD results in a higher burden for caregivers, associated with higher levels of behavioral disturbances in patients (5).

**Figure 5:**
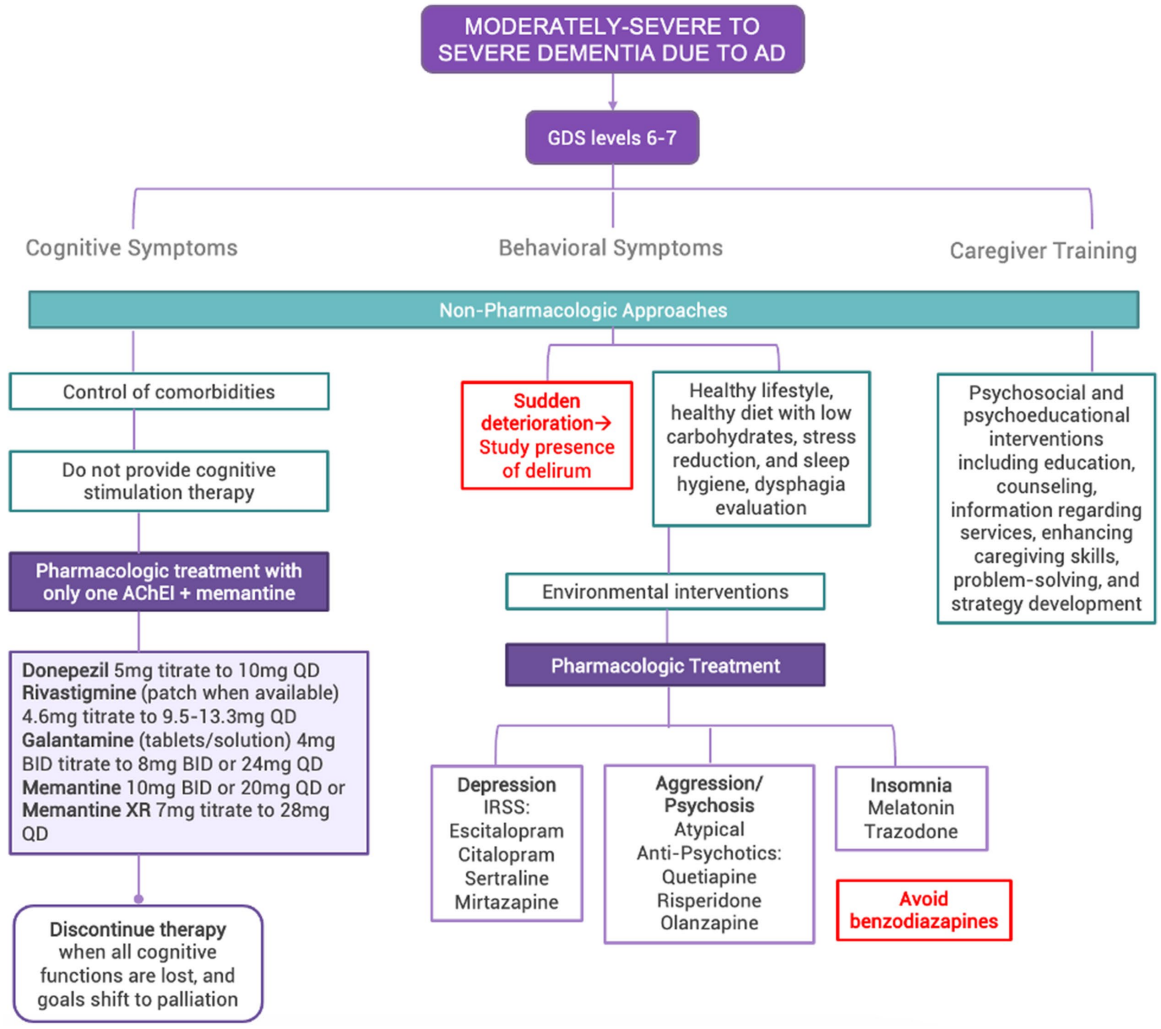
Algorithm for the management of moderately-severe to severe dementia due to AD in LAC. GDS, Global Deterioration Scale; CDR, Clinical Dementia Rating Scale; AD, Alzheimer’s Disease; AChEI, acetylcholinesterase inhibitors.

This group requires treatment and management based on the following principles (See Figure 5) and guidelines (Table 4).

**Table 4:** Summary of clinical practice guideline recommendations for the treatment of severe AD.

Condition	EAN Guidelines (44)	APA Guidelines (43)	CCCD Recommendations (42)
Severe AD	AChEIs and memantine. Consider expected benefits, potential side effects, and impact of expenses.	Modest evidence for the efficacy of AChEIs or memantine. Slight or unclear clinical significance for the combination therapy.	A combination of AChEI and memantine is rational and safe. Insufficient evidence to recommend for or against the combination.
Depression	SSRIs. Avoid tricyclic antidepressants.	Mixed evidence for the efficacy of antidepressants.	A trial of an antidepressant could be considered.
Agitation/Psychosis	Non-pharmacologic measures. Antipsychotic for severe behavior causing significant distress that has not responded to non-pharmacological actions.	Antipsychotics provide weak benefits. Tapered.	Antipsychotics (risperidone, olanzapine, aripiprazole) are recommended for severe agitation, aggression, and psychosis. Potential benefits must be weighed against the significant risk of stroke and mortality.

EAN, European Academy of Neurology; APA, American Psychiatric Association; CCCD, Canadian Consensus Conference on Dementia, AChEI, cholinesterase inhibitor. The latest recommendation by the American Academy of Neurology was published by Doody et al. (95), before the approval of memantine or memantine/donepezil.

Enteral nutrition (nasogastric tubes and percutaneous endoscopic gastrostomy) and hydration should be considered medical treatments rather than basic care and, therefore, should only be used if there is a realistic probability of improving or maintaining the patient’s condition and QoL. Enteral nutrition should not be initiated when no functional patient benefits are expected. Especially in patients where death is imminent (e.g., within the next 4 weeks or in advanced dementia), the patient’s comfort is the highest priority (106).

##### 3.8.4.1. Non-pharmacological treatment

Cognitive stimulation is ineffective at this stage (29). Activities should be restricted when patients can no longer participate safely and productively (107). Non-pharmacologic approaches should be considered in treating behavioral symptoms before pharmacologic ones (108, 109). Specific behavioral management can help reduce depression, aggression, incontinence (24), and insomnia (110).

##### 3.8.4.2. Pharmacological treatment for cognition

AChEI, combined with memantine, can be prescribed to these patients (101, 111–113). Several considerations must be made when deciding whether to continue treatment with the aforementioned depending on the clinician’s judgment. Because many LAC families must incur out-of-pocket expenses to acquire these therapies, physicians must evaluate the cost–benefit of using these medications related to the coverage of other needs in patients with severe AD (114, 115). Comorbidities must also be considered. Additionally, these treatments should not be initiated at a severe AD stage if the patient has not received them previously. Expected benefits include modest improvements or a slower decline in cognition function and behavior in severe AD (6, 116). The addition of memantine to AChEI significantly delayed nursing home admissions (117). Discontinuation of AChEI may worsen cognitive, neuropsychiatric, or functional status (118). Treatment should be continued until clinical benefit can no longer be demonstrated. Patients who are bedridden, noncommunicative, and can no longer perform basic daily activities can generally be safely withdrawn from medications. Medications should not be suspended simply because the patient has been admitted to a long-term care facility (1).

##### 3.8.4.3. Pharmacological treatment of behavioral and psychological symptoms

Severe behavioral and psychological symptoms (agitation, aggression, and psychosis) are the hallmark of severe dementia (5). However, the onset of *delirium* caused by infection, pain, other medical conditions, side effects from medication, and psychosocial or environmental factors must be considered in patients with a sudden worsening of behaviors (119, 120). Atypical antipsychotics and antidepressants may be initiated at the lowest doses, titrated slowly, and fully monitored (1) due to the anti-muscarinic adverse effects of these drugs (20). Attempts to taper or withdraw should be made (1) if there is no clinically significant response after 4 weeks (121) or (2) after 4 months of behavior stability (34). Risperidone, olanzapine, and quetiapine can be used for severe agitation, aggression, and psychosis. The potential benefit of all antipsychotic agents must be weighed against the potential risks, such as cerebrovascular events and death (122, 123). The mortality rate varies between first and second generation (haloperidol 20%, olanzapine 13%, risperidone 13%, quetiapine 9%) (34). Selective serotonin reuptake inhibitors can be used to treat severe depression (122). Benzodiazepines should be avoided or used in emergencies only for brief periods due to potential or paradoxical adverse events (falls, excessive sedation, worsening cognition) (20). Periodic medication review and de-prescribing opportunities guided by function and active life expectancy should be frequent practices in people with AD, especially those with multimorbidity (109, 124, 125).

##### 3.8.4.4. Caregiver training

Psychosocial and psychoeducational interventions for caregivers of people with AD should be provided (42). These can include education, counseling, information regarding services, enhancing caregiving skills, problem-solving, and strategy development. Some evidence-based health promotion programs can help care for persons with chronic diseases and caregivers’ self-care and self-efficacy (126).

##### 3.8.4.5. Physical environment intervention/ long-term institutional care

Physical environment interventions are beneficial. These include more visible toilets, outdoor areas with therapeutic design features, high light levels, moderate stimulation, and reduction of overstimulation when bathing (127). Most patients with severe dementia require institutional care; however (7, 8), families often decide to keep the patient at home in LAC (17, 21). This may result from caregivers’ guilt or, more frequently, a lack of coverage by the healthcare system and the family’s inability to cover these expenses (17). In the terminal phase of dementia, palliative care may be helpful (38).

### 3.9. Looking to the future

Disease-modifying AD treatments are currently being studied. More than 800 medications have been evaluated in clinical trials in recent years with negative results. This failure is mainly because they have been tested in the dementia stage, where the brain has suffered substantial damage, and it may be too late to obtain positive results. Recently, the strategy has begun to change, seeking to prevent or to clinical progression rather than cure the disease. LAC must prepare for the imminent paradigm shift for AD management from reactive treatment to prevention/disease modification.

Delaying the onset of AD symptoms by 5 years would reduce the prevalence and costs of the disease by 50% (128). Tertiary prevention consists of treating symptomatic people with MCI to avoid progression into dementia. Secondary prevention consists of treating asymptomatic people with neuropathological alterations of AD to prevent progression to MCI and, ultimately, to dementia. Primary prevention consists of treating asymptomatic people without neuropathological disease changes to avoid or delay it. In tertiary prevention, various anti-amyloid agents have been unsuccessful at slowing cognitive decline despite demonstrating biological improvement (129). Some anti-amyloid agents are evaluated in secondary prevention research, while primary prevention studies are planned for clinical trials. Lecanemab was associated with less cognitive and functional decline than placebo at 18 months in patients with MCI and mild dementia due to AD but was related to adverse events (130). Of note, the clinical significance of this change has been questioned (130). The drug has been recently approved for clinical use by the United States Food and Drug Administration and raises expectations for treating AD in its early stages. However, its prescription should still be limited to tertiary care centers, and its effect to be proven in clinical practice. The United States Food and Drug Administration has also approved Aducanumab. It has demonstrated biological but not clinical efficacy (131).

New alternatives with anti-tau, anti-inflammatory medicines, and combined treatments are also under development. However, in current clinical practice, only symptomatic treatment can be offered to patients with dementia, and tertiary prevention treatments to patients with MCI to prevent progression to dementia. Additionally, current research indicates that an anti-amyloid alone will not be enough to treat or prevent Alzheimer’s.

An understanding of AD genetics is necessary to understand potential future treatment targets. In this sense, there are two main variants of AD: familial AD, which has an autosomal dominant inheritance pattern and manifests early, before the age of 65, and sporadic AD, which is polygenic with a complex pattern of inheritance of susceptibility genes and is characterized by its late onset, after age 65.

Approximately 1–2% of AD cases are familial with Mendelian inheritance. The genes involved in the autosomal dominant familial pattern are known as causative genes because the carriers of some of these gene mutations have a 100% risk of developing the disease, given that these mutations have 100% penetrance. In the sporadic form, many susceptibility genes exist, which do not cause the disease but contribute to its risk. Systematic meta-analysis suggests that at least 20 loci have modest but significant effects on AD risk. The presence of *APOE* allele e4 is the most significant genetic risk factor in sporadic AD. Carriers of the e4 allele have a three to tenfold increased chance of developing AD. Other relevant susceptibility genes are clusterin (*CLU*), also known as *APOJ*, *PICALM*, *TOMM40*, *CR1*, and *LRP1* (132).

Autosomal dominant forms of AD are most frequently caused by mutations in the *PS1* gene (133). Through monitoring the population with the Paisa mutation (E280A in *PS1*) in Colombia, it has been possible to describe the pre-dementia stages of AD and its ages of onset. Moreover, it enabled the identification of a woman who carried both a causative mutation for AD and a protective mutation, delaying the onset of the disease for almost three decades. This patient, as a carrier of mutation E280A in *PS1*, would typically be condemned to the onset of symptoms of memory loss at the age of 44; however, she was homozygote for the Christchurch mutation (R136S) in *APOE3*, which caused the onset of MCI to be delayed to age 72. She had massive amyloidosis, low levels of tauopathy, and mild neurodegeneration. The effect of the protector mutation in weakening the ApoE-HSPG bond and reducing the dissemination of the tau protein could have therapeutic potential (9, 134).

We characterized the world’s second case with ascertained extreme resilience to autosomal dominant Alzheimer’s disease (ADAD). It was a man that remained cognitively intact until 70 years of age despite carrying a *PSEN1*-E280A mutation. Like the *APOECh* carrier, he had an extremely elevated amyloid plaque burden and limited entorhinal tau tangle burden. He did not carry the *APOECh* variant but was heterozygous for a rare variant in *RELN* (H3447R, termed Reelin-COLBOS). *RELN-COLBOS* is a gain-of-function variant that reduced human Tau phosphorylation in a knockin mouse. This genetic variant in a case protected from ADAD suggests a role for *RELN* signaling in resilience to dementia (135).

Box 1 **The three types of genetic biomarkers related to AD.**

Causation Biomarkers: Mutations in PPA, PS1, and PS2 that cause familial AD with early-onset before age 65, with an autosomal dominant Mendelian inheritance pattern.

Susceptibility Biomarkers: A range of genes or genetic variants confer susceptibility but do not cause the disease. The most important of these include the e4 variant of APOE and TREM2.

Protective Genetic Biomarkers: Genes or variants that have a protective effect, preventing or delaying disease onset. Includes the e2 variant of APOE (136), mutation A673T in PPA (137), the Christchurch mutation (R136S) in ApoE3 (138), the V236E variant in 22 APOE 3, and the R251G variant in APOE 4 (139). Reelin-Colbos mutation (H3447R) (135).

Summarizes causation, susceptibility, and protective biomarkers.

The AT (N) research framework recognizes three general groups of biological markers of AD based on the nature of the pathological process measured by each of them: A–amyloid, T–tauopathy, and N–neurodegeneration (1).

Box 2 **The three general groups of biological markers for AD.**

Biomarkers of cerebral amyloidosis (A): Amyloid-PET is a direct imaging biomarker of the presence of amyloid in the cerebral parenchyma, while the level of Aß-42 or the Aß-42/Aß-40 ratio in CSF is an indirect biochemical marker of the presence of Aß-42 deposits in the brain.

Biomarkers of cerebral tauopathy (T): Direct imaging method is PET-tau, which is a direct imaging biomarker of the presence of tauopathy in the brain. An indirect method is the level of phosphorylated tau (p-TAU) in the CSF. Plasma levels of p-tau217 and p-tau181 appear to have a promising future as low-cost and easy-to-access biochemical markers of tauopathy associated with AD (140).

Biomarkers of neurodegeneration (N): Brain MRI–direct imaging indicator of the degree of neurodegeneration and cerebral atrophy, measurable by cortical and hippocampal volumetry. PET with fluorodeoxyglucose (FDG) is a direct imaging biomarker of cerebral metabolism and an indirect, non-specific marker of neurodegeneration.

The exact alteration times of the different biomarkers in sporadic Alzheimer’s are not known, but in the autosomal dominant familial form with the E280A mutation in PS1 it has been discovered that carriers of the Paisa mutation show high levels of biomarkers such as ß_42_ in cerebrospinal fluid, NFL and phTAU in plasma at 24, 20 years before the first symptoms of Mild Cognitive Impairment. Additionally, neuroimaging studies have revealed that these individuals showed PET-amyloid positivity at 28 and pTAU positivity 10 years later at 38. Biomarkers of amyloidosis do not correlate well with alterations in cognition, but Imaging biomarkers of tauopathy and neurodegeneration correlate well with the onset of subjective memory complaints at 38 and later with mild cognitive impairment at 44 ad dementia at 49. Regarding cognitive markers, decreased performance in the word-list test of CERAD battery around 32 years is the earliest sign of cognitive decline. Currently, new research using digital neuropsychology tools aims to investigate the suitability of performance in spatial navigation tasks as cognitive markers since the preclinical stage of AD. This information is crucial for designing primary and secondary prevention clinical trials for Alzheimer’s.

To achieve a biological diagnosis of AD, it is necessary to demonstrate alterations in at least the biomarkers “A” and “T.” A person with abnormal changes in the biomarker “A” with normal “T” biomarkers cannot be diagnosed with AD but is said to have “pathological alterations of Alzheimer’s.” Nonetheless, “pathological alterations of Alzheimer’s” and “AD” represent phases on the same Alzheimer’s *continuum*. The “A” biological markers determine whether an individual is on the AD *continuum*. The “T” biological markers determine whether someone on the AD *continuum* has a neuropathological disease diagnosis. And the “N” biomarkers reflect the degree of atrophy from the clinical syndrome. Biomarkers and the ATN biological classification are primarily used to increase diagnostic accuracy (141). The AT(N) framework guides research on AD toward personalized medicine and allows for flexibility in the future regarding the addition of other biomarkers that may be discovered and validated.

## 4. Conclusion

LAC faces diverse challenges in the fight against AD. Regulatory entities and governments must encourage and facilitate global and regional AD research participation. Increased education and awareness efforts among the general population must be undertaken to dispel myths about memory loss. Creating public healthcare strategies to control modifiable risk factors for neurodegeneration and dementia syndrome in LAC could prevent up to 56% of dementia cases (19). Additionally, training at the primary care level is necessary to improve early AD detection and increase adequate referral situations. Because of the diverse populations in LAC, countries should have cognitive tests validated for their local context. A harmonized diagnostic approach using validated BCTs would increase early AD detection and provide the basis for research collaboration and standardized data generation throughout the region.

The financial burden of the direct and indirect costs related to AD care in LA are currently borne primarily by the families, exacerbated by minimal coverage of cognitive-enhancing medication through the public healthcare systems. Without access to improved treatments and preventive therapy, the adverse repercussions of AD will continue to impact LAC people, healthcare systems, and economies. Given that currently available biomarkers entail high costs and invasive procedures, there is an unmet need for peripheral biomarkers (plasma, saliva, tears, urine, fecal matter) for AD diagnosis that would provide a more accessible alternative to LAC economies and set the stage for prevention.

The high prevalence of genetic AD in Colombia and several other LAC countries suggests a similar situation throughout the region, necessitating dedicated search strategies for populations with genetic AD variants. Consequently, LAC has the potential to play an essential role in the future of primary and secondary prevention for AD.

We hope this document will be a tool to improve the expertise and professionalism of health professionals and thus optimize the individual care of people with AD and their families. And, importantly, it also equips professionals to be leaders in transforming health services in LAC countries (142, 143).

## Author contributions

FL, NC, RA, JB, EG, CC, AM, and SB: writing—original draft, investigation, formal analysis, and validation. AJ: writing—review and editing, methodology, and project administration. MR-R: writing—review and editing, visualization, conceptualization, methodology, and project administration. IC, PC, JD, RN, JP, CR, and AS: writing—review and editing, visualization, formal analysis, and validation.

## Funding

The organization and implementation of the consensus conference were carried out by the AHF, a 501(c)3 nonprofit organization dedicated to improving healthcare throughout the Latin American Region and was supported by an unrestricted grant from Adium. The funder had no influence on the design, implementation, and content of this manuscript.

## Acknowledgments

The authors would like to thank Ms. Thais Vidal, BA, for her assistance in language-editing the manuscript.

## Conflict of interest

The authors declare that the research was conducted in the absence of any commercial or financial relationships that could be construed as a potential conflict of interest.

## Publisher’s note

All claims expressed in this article are solely those of the authors and do not necessarily represent those of their affiliated organizations, or those of the publisher, the editors and the reviewers. Any product that may be evaluated in this article, or claim that may be made by its manufacturer, is not guaranteed or endorsed by the publisher.
